# Editorial: Regulation of Immune Function by the Lymphatic Vasculature

**DOI:** 10.3389/fimmu.2019.02597

**Published:** 2019-11-05

**Authors:** Beth Ann Jiron Tamburini, Timothy P. Padera, Amanda W. Lund

**Affiliations:** ^1^Division of Gastroenterology and Hepatology, Department of Medicine, School of Medicine, University of Colorado Anschutz Medical Campus, Aurora, CO, United States; ^2^Department of Immunology and Microbiology, School of Medicine, University of Colorado Anschutz Medical Campus, Aurora, CO, United States; ^3^Edwin L. Steele Laboratories, Department of Radiation Oncology, Massachusetts General Hospital Research Institute, Harvard Medical School, Boston, MA, United States; ^4^Department of Cell, Developmental, & Cancer Biology, Knight Cancer Institute, Oregon Health & Science University, Portland, OR, United States

**Keywords:** lymphatic vasculature, lymphatic endothelial cell (LEC), leukocyte trafficking, lymphedema, immune regulation, cancer lymphatics, antigen presentation

The lymphatic vasculature is composed of a hierarchy of vessels that extend from peripheral tissues into lymph nodes (LN) and provide the critical route for leukocyte migration and antigen presentation that drive innate and adaptive immune responses. However, beyond physically connecting peripheral tissue to LNs, there is an expanding view of how the lymphatic vasculature influences immune responses. The lymphatic vasculature can modulate immune responses ranging from peripheral tolerance during homeostasis to protective immunity following infection or vaccination to tumor immune escape. Recent work demonstrates that lymphatic vessels actively regulate transport functions, express and secrete chemo-attractants to initiate leukocyte migration, and directly interact with leukocytes to inform their behavior, activation, and establishment of memory. The immunological impact of how the lymphatic vasculature is influenced by tissue microenvironments and ongoing regional inflammatory processes is only beginning to be appreciated, but is of critical relevance to the role lymphatic vessels play in diseases. This Research Topic, “Regulation of Immune Function by the Lymphatic Vasculature,” brings together 17 articles that present our current understanding of and provide new insight into the necessary role the lymphatic vasculature plays in regulating inflammation and immunity.

Lymphatic vessels mediate the exit of multiple leukocyte types from peripheral tissue and LNs, including dendritic cells, neutrophils, T cells and B cells. A comprehensive review by Jackson presents the molecular mechanisms that determine leukocyte homing and transmigration to and through peripheral lymphatic vessels while Hampton and Chtanova describe the immunological consequences of leukocyte exit in disease. Farnsworth et al. complement these insights with an extensive review of the interplay between lymphatic vessels and chemokines, and how the dynamic display of various chemokine ligands in response to microenvironmental stimuli influences the migratory nature of leukocytes. Interesting new data presented by Campbell et al. describe novel MHC-dependent interactions between T cells and dendritic cells within peripheral lymphatic capillaries. These exciting data suggest that within lymphatic capillaries, dendritic cells may interact with circulating T cells prior to entry into the LN. How the kinetics of leukocyte egress from peripheral tissues contributes to disease remains a critical question moving forward and raises the possibility that lymphatic vessels may help to pattern both the initiation and resolution phases of peripheral tissue inflammation.

Given the multiple roles lymphatic vessels play in regulating leukocyte trafficking and function, the field has long considered how lymphatic function, or dysfunction, contributes to the perturbation of immune homeostasis. Intriguingly, lymphatic vessel expansion is associated with autoimmune, chronic inflammatory, and malignant contexts. However, it remains unclear whether lymphatic remodeling is a cause of or rather a biproduct of tissue inflammation. Schwager and Detmar provide an extensive review of the mechanisms that regulate inflammatory lymphangiogenesis and discusses implications for disease and Schwartz et al. review the current literature surrounding the contribution of lymphatic dysfunction to autoimmunity. Providing new insights, Stephens et al. demonstrate that TLR4 signaling during intestinal inflammation is required for mesenteric lymphatic vessel expansion and leakiness, which leads to associated changes in dendritic cell migration to draining LNs. Though inhibition of TLR4 signals restored lymphatic function, there was no change in tissue inflammation, suggesting lymphatic vessel dysfunction lies downstream of inflammatory insult. Interestingly, Tamburini et al. also describe disease-dependent changes in lymphatic endothelial cell (LEC) transcriptional states in human chronic liver disease. Whether these changes cause inflammation or are the response to inflammation remains to be carefully determined.

The interplay between lymphatic and immune dysfunction is perhaps most apparent in the context of lymphedema. As lymphedema progresses, tissue fluid accumulation, and stasis cause multiple tissue changes, including fibrosis, adipose tissue deposition, and chronic inflammation. These tissue changes, along with impairment in the ability to transport antigen and antigen presenting cells to lymph nodes, drives progressive deterioration in local immune function. Kataru et al. and Yuan et al. provide back to back reviews highlighting the cellular and molecular mechanisms that occur during lymphedema and their impact on immunity in mouse models and outcomes for patients.

In this Research Topic, and in the field in general, much emphasis is placed on peripheral lymphatic vessel remodeling, however, Lucas and Tamburini discuss the molecular mechanisms of LEC expansion and contraction in LNs draining inflamed tissues and the role of LN LECs in regulating antigen presentation vs. antigen exchange. Louie and Liao further review the role of subcapsular macrophages in the LN, whose position and survival depend on LN LECs, in innate pathogen defense. Interestingly, the LN LEC population may also be altered in the context of chronic disease. Tay et al. present new data that describes reduced lymphocyte egress from LNs during hypercholesterolemia, which results in LN hypertrophy. These changes in lymphocyte trafficking were likely driven at least in part by altered CCL21 and S1P gradients, signaling molecules generated by LN LECs.

LECs, particularly those found in LNs, were recently demonstrated to harbor intrinsic antigen-presentation capabilities and to archive antigens in order to influence T cell activation. Antigen-dependent and independent interactions between LECs, T cells and DCs may both preserve peripheral tolerance and promote protective immunity depending on context. These observations have continued to fuel the concept that LECs directly influence immunity. This hypothesis is now further supported by transcriptional data from Berendam et al. that describe modules of immune transcripts within LN LECs that support the novel functionality of LN LECs and provide the basis for future mechanistic studies. These data are summarized and integrated with the existing knowledge by Santambrogio et al. in order to describe the machinery found within LECs that may facilitate antigen scavenging and presentation.

Finally, the contribution of lymphatic vessels to tumor progression remains an area of intense interest. While historical paradigms associate lymphatic vessels with regional tumor metastasis, new work integrates the potential effects of lymphatic vessel remodeling on anti-tumor immunity and immunotherapy. Garnier et al. provide a comprehensive review of the existing framework for lymphatic vessel involvement in anti-tumor immunity, describing both the impact of lymphatic vessel remodeling on immunity and responses to local inflammation. Tamburini et al. demonstrate that the microenvironment of post-partum mammary gland involution potentiated immunotherapy leading to reduced tumor volume, increased immune cell activation and decreased lymphatic vessel density compared to tumors in nulliparous hosts. Lymphatic vessels are high expressors of PD-L1 in involuting mammary glands and may contribute to this response, consistent with recent published data also demonstrating a functional role for PD-L1 on tumor-associated lymphatic vessels.

With this cumulative work, our field continues to show that lymphatic vessels are active components of host immunity. The mechanisms through which lymphatic vessels synchronize immune expansion and contraction in both LNs and peripheral tissues provide therapeutic opportunities for immunomodulation and will ultimately make a significant impact on our understanding of health and disease.

## Author Contributions

All authors listed have made a substantial, direct and intellectual contribution to the work, and approved it for publication.

### Conflict of Interest

The authors declare that the research was conducted in the absence of any commercial or financial relationships that could be construed as a potential conflict of interest.

